# Draft genome sequence of *Planococcus sp*. SK3692

**DOI:** 10.1128/mra.00545-23

**Published:** 2023-12-20

**Authors:** Sophie M. Kiehl, Riley Anderson, Traci L. Kinkel, Mark D. Stenglein

**Affiliations:** 1 Department of Microbiology, Immunology and Pathology, Colorado State University, Fort Collins, USA; University of Strathclyde, Glasgow, United Kingdom

**Keywords:** bacteriology, genomics, *Planococcus*

## Abstract

Whole-genome sequencing was performed for a *Planococcus sp*. isolate. This bacterium was of interest because of its vibrant orange pigmentation.

## ANNOUNCEMENT

This bacterium was isolated in January 2021 as part of the Small World Initiative (1) from the brushed fur of a dog that had recently been in the Pacific Ocean at Camarillo, CA (34.2164N 119.0376W). The sample collection protocol (#5131) was reviewed by Colorado State University’s Institutional Animal Care & Use Committee Waiver Subcommittee and determined to be an exempt activity. Fur was placed in phosphate-buffered saline and a dilution series was plated on tryptone soy agar (TSA). Plates were incubated for 5 days at 30°C aerobically. An orange-pigmented colony was subsequently restreaked on TSA until a pure isolate was obtained.

An overnight tryptone soy broth culture was inoculated with a single colony and grown aerobically at 30°C. DNA was extracted from the culture using the QiaAmp Fast DNA Tissue Kit (Qiagen; Valencia, USA) following the manufacturer’s protocol with a lysozyme pre-incubation step. This DNA was input to Illumina and nanopore sequencing. For Illumina library preparation, DNA was fragmented using a Covaris M220 ultrasonicator to an average fragment length of ~400 bp. DNA size distributions were assessed using Agilent 2200 Tapestation. Illumina libraries were prepared using a sparQ DNA library prep kit (Quantabio; Beverly, USA) following the manufacturer’s protocol. Libraries were size-selected to a range of 300–600 bp using a BluePippin instrument (Sage Science; Beverly, USA). Libraries were sequenced on an Illumina MiSeq instrument using a v2 500 cycle sequencing kit to generate 2 × 250 paired-end reads.

Cutadapt 3.5 (2) removed adapter sequences, reads shorter than 80 bases, and bases with quality score less than 30. This reduced 1,688,050 Illumina reads to 1,636,878.

Nanopore libraries were prepared using the Rapid Barcoding Kit SQK-RBK004 [Oxford Nanopore Technologies (ONT); Oxford, UK] following the manufacturer’s protocol using unfragmented input DNA. Libraries were sequenced on a MinION device using one ONT Flongle R9.4.1 flow cell (FLO-FLG001). Basecalling was performed with guppy 6.3.4 (3). The 6045 nanopore reads had an average length of 6312 bp, and no reads were filtered by length or quality (4).

Long and short reads were assembled using SPAdes 3.15.5, with contigs scaffolded using long reads (5). In general, software used default parameters; full command lines are available in the github repository linked below. Assembly statistics were calculated using Quast 5.2.0 (6). The assembly contained six scaffolds longer than 400 bp (lengths 1.8 Mb, 1.5 Mb, 234 kb, 48 kb, 12 kb, 7 kb). A BLASTN 2.13.0 (7) search of scaffolds against the NCBI nucleotide database returned *Planococcus sp*. sequences with alignment identities of ~90%, with the largest three scaffolds aligning to chromosomes and the shorter three aligning to plasmid sequences. The assembly was 3.5 Mb long and had an N_50_ of 1.8 Mb and a GC content of 47.5%. The average coverage depths of 108× and 10.5× for Illumina and nanopore reads, respectively, were calculated by mapping with minimap2 v2.2.4 and using samtools v1.17 (8, 9).

The GenBank assembly was annotated using PGAP 6.4 (10), which identified 3,563 genes (3,450 protein-coding). NCBI’s automated taxonomic assignment verification, based on average nucleotide identity, corroborated the *Planoccocus* genus assignment (11).

## Data Availability

Assembly ASM2889048v2 has been deposited in DDBJ/ENA/GenBank under BioProject accession PRJNA929260, BioSample accession SAMN32954226. Raw reads have been deposited in SRA under accession SRS16684895. The accessions for the Illumina and Nanopore reads are, respectively, SRX19283240 and SRX19283241. The analysis code is available at GitHub.
